# Volatile Organic Compounds in Air: Sources, Distribution, Exposure and Associated Illnesses in Children

**DOI:** 10.29024/aogh.910

**Published:** 2018-07-27

**Authors:** Regina Montero-Montoya, Rocío López-Vargas, Omar Arellano-Aguilar

**Affiliations:** 1 Instituto de Investigaciones Biomédicas, Universidad Nacional Autónoma de México, MX; 2 Facultad de Ciencias, Universidad Nacional Autónoma de México, MX

## Abstract

**Background:**

Toxic volatile organic compounds (VOC), like benzene, toluene, ethylbenzene and xylenes (BTEX), are atmospheric pollutants representing a threat to human health. They are released into the environment from mobile sources in urban settings, but newly polluted areas are gaining importance in countries where accelerated industrialization is taking place in suburban or rural settings.

**Methods:**

The review includes studies done in Mexico and Latin-America and countries considered to have emerging economies and are compared with similar studies in developed countries. Data about environmental VOC levels and exposure of children have been included. Also, information about health effects was reviewed. Articles were searched in PubMed and Scopus, and information was also obtained from the United States Environmental Protection Agency (EPA), the EPAs Integrated Risk Information System (IRIS-EPA) and state reports on air quality of Mexican cities.

**Results:**

VOC or BTEX levels reported in industrial and suburban areas were found to be higher due to the burning of fossil fuels and waste emission; whereas, in big cities, VOC emissions were mainly due to mobile sources. Even though TEX levels were under reference values, benzene was found at levels several times over this value in cities and even higher in industrial zones. Elevated VOC emissions were also reported in cities with industrial development in their peripheral rural areas.

**Public health relevance:**

Industrial activities have changed the way of life of small towns, which previously had no concern about environmental pollution and chemicals. No air monitoring is done in these places where toxic chemicals are released into rivers and the atmosphere. This work demonstrates the need for environmental monitors to protect human life in suburban and rural areas where industrial growth occurs without planning and ecological or health protection, compromising the health of new generations beginning in fetal development.

## Introduction

Environmental pollution is a significant problem for humanity because of considerable changes in ecosystem behavior and the loss of biodiversity it is triggering, and because it may be at the origin of different diseases and physiological disorders in humans. Pollutants that impact air quality include volatile organic compounds (VOCs), which are introduced into the atmosphere through anthropogenic or biogenic activities and add to problems in the formation of tropospheric ozone and particles lower than 2.5 micrometers in large cities [1].

VOCs as categorized by the World Health Organization (WHO) are compounds with a boiling point less than 250°C measured at a standard atmospheric pressure of 101.3 kPa [2]. This classification may be further subdivided into very volatile organic compounds with boiling points between 0 and 100°C, which are mainly gases, and volatile compounds with boiling points between 100 and 250°C, distributed between air and water body surfaces or solid surfaces [3]. These compounds’ physical and chemical properties and mean lifetime in the atmosphere, which ranges from a few minutes to several months, allow them to travel large distances from the source of emission and to enter the body, mainly by way of air or through the skin, and cause symptoms that may lead to pathologies, including asthma, atopic dermatitis and neurologic problems. Some VOCs, like benzene, 1,3-butadiene and vinyl chloride are classified by the International Agency for Research on Cancer (IARC) in Group 1 as carcinogenic for humans [4].

Nevertheless, these data were obtained from occupational studies, so contribution to the aforementioned diseases by environmental exposure to VOCs is generally unknown, with the best documented being respiratory diseases caused by ozone [5]. VOCs, however, are important for global ecological integrity and human health [6] independently of their contribution to tropospheric ozone. In Mexico, information about VOC mixture in air is scarce, and the most widely studied sites are large cities, like Mexico City (MX), Monterrey (MY) and Guadalajara (GJ); industrial areas have been studied only in relation to aromatic VOC levels, such as benzene, toluene, ethylbenzene and xylenes (BTEX), as surrogates for the presence of VOC, because they result mostly from fuel combustion.

Therefore, in this review we first compared VOC levels in the three above-mentioned cities as to their composition and levels. Next, we focused on identifying the value of BTEX in the environment in communities close to industrial facilities from which these substances may be released, and we focused on any relationships that may exist under child population exposure conditions. These data were used to make a comparison with occupational exposure reference values. The purpose of this review was to describe the information currently available about VOC toxic effects and VOC levels in our cities and their industrial areas so as to better understand the problems faced by communities more affected by these toxic substances.

## Methodology

Works between 2000 and 2017 were reviewed in PubMed and Scopus following searches with the key words volatile organic compounds, health, disease and oxidative stress. Also, the latest information from the EPA and IRIS was reviewed and information about BTEX was used as a model and summarized. Public records from the National Institute of Ecology and Climate Change (INECC in Spanish) were consulted for information about benzene levels in Mexican Cities.

## Results

### Volatile organic compounds (VOCs)

VOCs include aromatic hydrocarbons, aliphatics, aldehydes, ketones, ethers, acids and alcohols, with diverse functional groups (halogens, oxygen, sulfur, nitrogen or phosphorus, excluding carbon oxides and carbonates). They are rather inert lipophilic compounds capable of passing through biological membranes, with a toxicity that basically depends on their biotransformation within the body [7]. Although a large number of substances are considered VOCs, the most abundant in the environment are benzene and some of its organic derivatives, like toluene, ethylbenzene and xylene (o-, m- and p-), jointly named BTEX, which comprise over 60% of the VOCs found in urban areas [8]; hence, they are used as a reference to evaluate environmental levels and VOC exposure.

VOCs come mainly from natural sources, like forest fires and the transformation of biogenic precursors; nevertheless, anthropogenic activities have become important sources of toxic VOC emissions into the atmosphere, so much so that they account for 25% of VOCs in our global atmosphere [9]. Petroleum and natural gas extraction, petrochemical activities and the burning of fossil fuels in industries, homes and mobile sources, including automobiles, trucks, buses and motorcycles, ships and airplanes are the major contributors of VOC, followed by chemical and industrial processes (manufacturing of paints, lubricants, adhesives, oil derivatives), mining, commercial sources, gas leaks from stoves, residential water heaters and boilers and use of pesticides in agriculture [10,11].

To determine the amount of pollutant emissions into the atmosphere, the US Environmental Protection Agency (EPA) developed the national emissions inventory (NEI), where a comprehensive and detailed estimate of air emissions of the most hazardous atmospheric pollutants and their precursors is made from different sources across its territory [12]. Pollutants included in the NEI are associated with the national ambient air quality standards (NAAQS) and with the EPAs air toxic program, which include hazardous air pollutants (HAPs). NEI estimates of compounds include carbon monoxide (CO), lead (Pb), particulate material (PM), sulfur dioxide (SO_2_) and ozone precursors (O_3_), which are nitrogen oxides (NO_x_) and VOCs.

Regarding VOC emissions from industrial processes, the EPA reported in 2014 the activities with the highest VOC emissions into the atmosphere were oil and gas production, totaling more than 3 million tons that year, followed by storage and transportation, and new and existing chemicals (NEC) with nearly 200 thousand tons in both cases; whereas, oil refineries emitted just over 50 thousand tons [13]. It is worth mentioning that these figures account for only the United States, and so it is of concern that not all countries have reliable air pollution emission inventories, because despite the existence of the Global Atmosphere Watch (GAW), it monitors only VOCs present in large geographic areas, not at a regional municipal level, where health impacts occur.

Table 1 presents VOC pollution compositions and levels from a study on air quality in major cities in Mexico [14,15,16]. In MX, the composition is due to liquified gas, automobile and truck fuel combustion and the use of household products of industrial origin besides fuel combustion. In MY, a substantial contribution of compounds of industrial origin besides fuel combustion is observed; levels reached in the most polluted area in MY (Santa Catarina) are about twice the highest levels measured in MX (La Merced): 1051 ppbV versus 605.5 ppbV [14,15]. GJ shows an intermediate combined composition of industry, gas and use of industrial products, with a total of 678.04 ppbV average [16].

**Table 1:** VOC mixture in the air of the largest cities in the Mexican Republic.

	Monterrey [15]			Guadalajara [16]			México [14]		

	VOC	Origin/use	Maximal ppbV	VOC	Origin/use	Maximal ppbV	VOC	Origin/use	Maximal ppbV

1	Ethanol	Industrial/fuel combustion	150	Propane	LP gas	160	Propane	LP gas	28
2	Propane	LP gas	150	Methylcyclo-pentane	Industrial	70	Acetone	Domestic product	17
3	2-hexanone	Industrial	105	n-butane	LP gas	38	Ethanol	Fuel combustion	15
4	Acetone	Industrial	60	Acetone	Domestic use	30	n-butane	LP gas	15
5	4-methyl-2-pentanone	Industrial/domestic product	40	Ethanol	Fuel combustion	30	Toluene	Fuel combustion	8
6	2-propanol	Industrial/domestic products	35	2-hexanone	Industrial	30	n-pentane	Fuel combustion/domestic product	10
7	Hexachloro-1,3-butadiene	Industrial	35	2-propanol	Industrial/domestic product	25	Isopentane	Fuel combustion/domestic product	8
8	1,4-dichlorobenzene	Industrial	30	Toluene	Fuel combustion/domestic product	20	n-hexane	Fuel combustion	6
9	1,2,4-trimethylbenzene	Fuel combustion/industrial	30	Isobutane	LP gas	18	Ethylene	Domestic product/fuel combustion	5
10	n-butane	LP gas	30	4-methyl-2-pentanone	Industrial/domestic product	18	Isobutane	LP gas	5
	TOTAL VOC	Santa Catarina area	1051	TOTAL VOC	Average Whole City	678.04	TOTAL VOC	La Merced area	605.5

TOTAL VOC – refers to the sum of all VOC measured; in the table, only the ten more abundant are presented.

The relative abundance of reactive VOCs that contribute to the formation of tropospheric ozone in MY and MX was studied [14,15], and it was found that the potential for ozone formation in the most polluted industrial area in MY was four times higher (Table 2) than the potential in the most polluted area in MX. Nevertheless, measurements in 2015 indicated higher ozone levels in MX (50 to 80 ppb during the ozone generation period, from March to June, with a maximal value of 179 ppbV in June, at the south of the City), which is probably because the natural atmosphere in MX is very oxidative and UV radiation is high [17]. MY showed ozone levels between 20 and 36.6 ppbV on average throughout the year, with a maximum 46 ppbV in August in its NW area [18]. According to the EPAs air quality indices (AQI) for ozone, MY shows good indices; whereas, MX shifts between moderate to harmful for sensitive groups values [19].

**Table 2:** Potential contribution of VOC to ozone formation according to their MIR* factors in Monterrey and Mexico City.

Monterrey (Santa Catarina area) [15]		Mexico City (La Merced area) [14]	

VOC	Potential ozone formation (ppbV)	VOC	Potential ozone formation (ppbV)

Ethanol	233.5	Toluene	72.8
Acrolein	132	o-xylene	42.8
Methyl-metacrylate	108.7	Ethanol	21.4
Toluene	101.0	Propylene	21.2
Propylene	82	Ethylene	20.8
1,2,4-trimethylbenzene	79.5	1,2,4-trimethylbenzene	17.5
o-xylene	79.4	m-xylene	14.5
m-xylene	78.4	n-hexane	11.9
p-xylene	71.8	Isopentane	11.7
1-pentene	64.9	n-butane	11.5
4-methyl-2-pentanone	59.2	1,3,5-trimethylbenzene	9.7
Propane	57.8	1,3-butadiene	9.7
Naphthalene	55.7	Propane	8.9
1,3,5-trimethylbenzene	50.9	p-xylene	8.7
n-hexane	50.3	Ethylbenzene	7.7
**TOTAL POF**	**1,305.1**	**TOTAL POF**	**290.8**

* Maximum Incremental Reactivity. POF – potential ozone formation.

### Benzene, toluene, ethylbenzene and xylenes (o-, m- and p-) (BTEX), as indicators of toxic VOC exposure

Benzene, toluene, ethylbenzene and xylenes (o-, m- and p-) are found in natural form in crude oil, diesel and gasoline, so they are released into the environment whether or not these fuels are burned. In addition, BTEX are highly used in the industry as additives and precursors of other substances: benzene is used in the manufacturing of synthetic materials and consumer products, including plastics, nylon, insecticides and paints; toluene is used as solvent for paints, coatings, rubbers, oils and resins; ethylbenzene may be found in paints, plastics and pesticides, and it is also used as additive for aviation fuel; xylenes are used as solvent in the printing, rubber and leather industries [20,12].

Because BTEX are so widely used and are derived from gasoline and organic material combustion, they coexist as a mixture in the atmosphere in practically all ecosystems. The mixture composition varies and depends to a great extent on nearby sources, on the type of industry, on the activities developed in the region and on environmental conditions. These compounds may be found polluting not only the air but also the soil and the water, because, as already mentioned, their physical and chemical properties endow them with a great dispersion capacity, and once they are released into the environment, they can volatilize, dissolve in water, adhere to soil particles or be biologically degraded [21,10]. High BTEX concentrations have been found in areas with intensive industrial activity, but they also reach high levels in large cities, mostly owing to vehicular traffic problems [22,21]. Furthermore, there may be additional exposure owing to household products, like disinfectants, aerosols, varnishes, printing inks [23], as well as cigarette smoke, which turns them into ubiquitous pollutants. Higher BTEX concentrations have been reported in close spaces (i.e., indoors) than in open spaces [10], but the ban on indoor smoking and more careful measurements [24,21] have demonstrated that personal exposure to VOCs may be due to outdoor pollutants, mainly in temperate and warm latitudes where doors and windows are opened more frequently, allowing for an air exchange. In MX, only toluene showed higher levels indoors than outdoors (26.11 ppbV vs 14.32 ppbV) [24].

For different polluting compounds in environmental matrices, the EPA has used information from the IRIS database to establish reference concentrations (RfCs) that cause critical effects [25]. Hence, the maximum environmental exposure concentration in air of benzene for an individual is 0.03 mg/m^3^; this concentration is due to the hematological effect of benzene in humans. For toluene, it is 5.0 mg/m^3^, based on neurological effects in humans and the increase in liver size. For ethyl-benzene, it is 1.0 mg/m^3^ based on effects in the respiratory system, hepatotoxicity and nephrotoxicity. For xylene mixture, a maximum concentration in air of 0.1 mg/m^3^ was estimated for effects on the respiratory and neurological systems and low birth weight (Table 3, bottom) [25]. All of the above refers to noncarcinogenic effects. Nevertheless, according to the monograph on specific reference values for benzene, an individual’s chronic exposure to a reference value of 0.03 mg/m^3^ accounts for an increase in the potential risk for developing leukemia to 1 out of 10, 000, compared with a baseline of 1 in one million for a population with exposures 2 order of magnitude lower [25].

**Table 3:** Concentrations of BTEX in different studies (µg/m^3^).

*Benzene µg/m^3^	Toluene µg/m^3^	Ethylbenzene µg/m^3^	o-xylene µg/m^3^	m, p-xylene µg/m^3^	Country	Type of measurement	Reference

**1.21**	14.33	2.55	2.16	5.97	USA population	Personal exposure	[66]
0.63	1.09	0.32	0.26	.	Canada	Outdoor levels	[67]
0.78–0.88	.	.	.	.	Stenungsund, Sweden, Petrochemical area	Outdoor levels	[68]
**2.15**	6.83	1.28	1.46	3.56	USA population	Outdoor levels	[21]
**3.64**	19.2	2.78	2.87	8.07	USA population	Personal exposure	[21]
**1.5–6.95**	7.17–26.9	0.59–2.06	0.94–4.16	3.07–13.3	Review of studies in the world	Outdoor levels	[10]
**1.21–2.8**	14.33	2.55	2.16	5.97	Review of studies in the world	Personal exposure	[10]
**15.07**	139.35	24.68	13.39	27.88	Kwai Chung in Hong Kong industrial area	Outdoor levels	[8]
0.7**–3.5**	2.3–6.0	0.4–5	.	1.9–2.3	Viseu, Portugal	Outdoor levels	[40]
**13.42**	18.9	1.8	2.3	10.91	La Plata industrial area, Argentina	Outdoor levels	[69]
0.58–**3.0**	2.8–5.9	0.2–1.6	0.26–1.3	1.3–3.5	Curitiba, Brazil, suburban area	Outdoor levels	[70]
0.58**–6.0**	4.3–73	0.19–2.5	0.24–45	1.3–6.9	Curitiba, Brazil, suburban area	Personal exposure	[70]
**5.9**	37.9	5	5.9	14.9	Mexico City	Outdoor levels	[24]
**10.6**	86.1	8.1	9.1	25.2	Mexico City	Personal exposure	[24]
**2.18–3.7**	17.17–46.9	2.4–7.2	2.8–11.3	3.8–11.7	Mexico City	Outdoor levels	[17]
**1.1–5.3**	2.3–14.0	0.4–2.2	0.5–3.2	1.4–8.0	Industrial area, Tlaxcala, Mexico	Outdoor levels	[74]
*0.03*	*5*	*1*	*0.1*	*0.1*	*USA*	*Outdoor levels*	*Rfc (mg/m^3^)* [71]
*1.01*	*6.95*	*1.5*	*1.5*	*4.1*	*Global*	*Personal exposure*	** µg/m^3^

* Bold numbers in the benzene column represent increased risk of leukemia for those populations. ** Lowest concentrations found to produce health effects [10].

**Figure 1:**
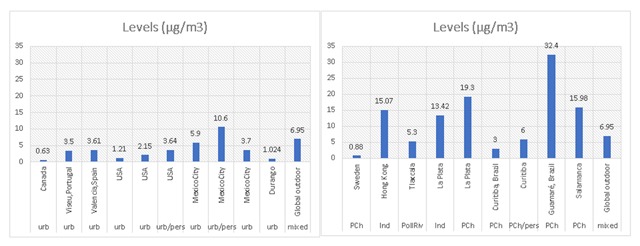
Highest levels of benzene reported in urban and industrial areas, both in developed and developing countries. Notice the extraordinary low levels reported in urban Canada (left panel) and industrial Sweden (right panel). Industrial areas in Latin America are not continuously monitored for air quality, only great cities, but children and pregnant women live in these places where pollution is generally high. Urb-urban; pers-personal; PCh-petrochemical zone; Ind-industrial zone; PollRiv-polluted river.

Table 3 also shows environmental exposure levels measured in research studies of populations near BTEX sources, like industrial areas, refineries and a highly polluted Mexican river. In all these cases, the reported concentrations were lower than RfCs set forth by the EPA for noncarcinogenic effects. However, most data reported about benzene—the only carcinogenic compound—are near or above the inhalation unit risk (IUR) value of 1 µg/m^3^ in the air, increasing an individual’s cancer risk by 2.2 × 10^–6^ up to 7.8 × 10^–6^ for being exposed for a lifetime. For example, the 15.07 µg/m^3^ value reported by Lee [8] would increase the cancer risk for that population 100 times [25]. As can be observed, industrial areas in emerging economies, like Latin-American countries and Hong Kong, show the highest levels of benzene, demonstrating that economic growth and development are not related to environmental protection and health or well-being (Figure 1).

### Biotransformation mechanisms and health effects of BTEX

**Figure 2:**
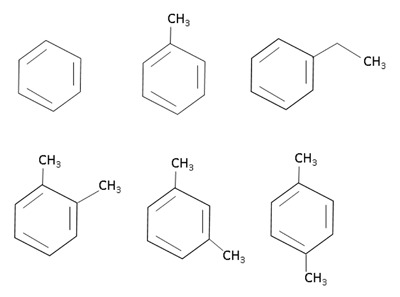
Chemical structure of BTEX. Upper line: benzene, toluene and ethylbenzene. Lower line: orto-, meta- and para-xylene.

BTEX compounds (Figure 2) are aromatic hydrocarbons derived from benzene; the toluene and ethylbenzene rings are monosubstituted with a methyl group and an ethyl group, respectively; the xylene ring is disubstituted with methyl groups, so that the xylene mixture comprises ortho-, meta- and para- (o-, m- and p-) compounds, according to the position of the ring substituents [26]. Because of these structures and their easy volatilization, they can enter organisms through various routes: the most common is inhalation, followed by skin and, at a lower percentage, orally through polluted water or food.

Toxicokinetic studies in humans and animals indicate that these lipophilic chemical products are distributed to highly vascular tissues rich in lipids, like the brain, the bone marrow and the body fat, and they are rapidly eliminated from the body. Nevertheless, such lipophilicity confers on them the capability to cross mucous epithelia of the respiratory tract and the cell membranes in various organs. The nose mucous epithelium and the tracheobronchial tree are metabolically active to BTEX exposure, although the liver is the organ with the highest activity, because most xenobiotic biotransformation processes take place in the liver [27].

BTEX are slightly reactive compounds, so their toxicity is determined by their biotransformation within the body [7]. Biotransformation comprises phase I and phase II processes.

Classical studies about BTEX biotransformation, like the toxicological profile for total petroleum hydrocarbons [28], considered that out of the four compounds, benzene has the highest toxicity potential because the formation of an epoxide was recognized from the oxidation of the benzene molecule by CYP2E1 action. However, more recent studies, such as those presented by the Agency for Toxic Substances and Disease Registry (ATSDR), reevaluated the joint toxic action of these chemicals, and now it is known that at low exposure concentrations BTEX are substrates of CYP2E1 and of other P450 isoenzymes, whose main function is to oxidize their substrates. Oxidized metabolites of this phase I reaction are highly reactive [20]. Furthermore, the action of compounds has a competitive metabolic inhibition mechanism, but only at high exposure levels; such mechanism has not been found at low levels. Hence, it was concluded that the biotransformation of the four substances is dose-dependent and generally extensive at dose levels that do not saturate the first metabolic step of each compound [29,20].

All four chemicals can produce neurological impairment via parent compound-induced physical and chemical changes in nervous system membranes. Additionally, exposure to benzene can cause hematological effects, including aplastic anemia, with subsequent manifestation of acute myelogenous leukemia via the action of reactive metabolites [30,20].

**Figure 3:**
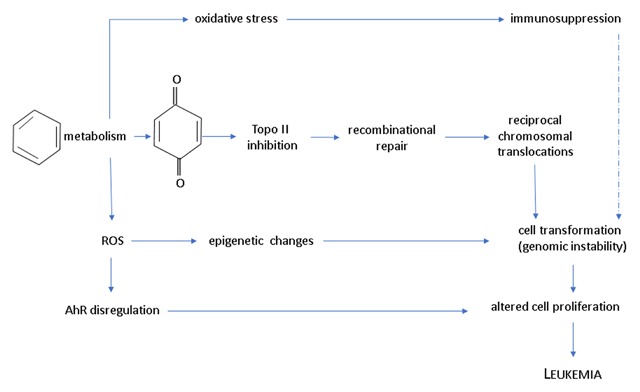
Probable pathways of benzene leukemogenic activity. These events might take place either in the fetal liver or in the infant bone marrow, or in both, to produce childhood leukemia (based on [10,56]).

Benzene, mainly during its metabolism, may suffer a nonenzymatic rearrangement and form phenol, which by a second CYP2E1-mediated oxidation turns into hydroquinone. This may be accumulated in the bone marrow and act as a substrate of myeloperoxidase (MPO), forming benzoquinone that is both myelotoxic and clastogenic [31]. Its leukemogenic capacity has partly been attributed to this mechanism (Figure 3). It has been determined that benzene may suffer biotransformation in the bone marrow, but in a lower quantity compared to the liver, although the typical toxicity site of this compound is the bone marrow [32,33]. At low doses, benzene turns into recognized toxic metabolites that include benzene epoxide or oxide, benzene dihydrodiol, hydroquinone, catechol, benzoquinones and muconaldehyde, while at a high dose, benzene inhibits hydroquinone formation from phenol, apparently through competition for the CYP2E1 active site, which also has an affinity for hydroquinone and catechol [31,33]. This is important because environmental exposure to benzene typically occurs at very low doses.

The American Conference of Governmental Industrial Hygienists (ACGIH) [34] made recommendations for the occupational biological exposure index (BEI®) in urine specimens for workers at industries producing environmental pollutants. In 2005, they recommended the use of certain metabolites to determine exposure to benzene and toluene, establishing that trans, trans-muconic acid (t,t-MA) was a good biomarker of benzene exposure with a BEI® of 500 µg/g of creatinine; for toluene, they established hippuric acid (HA) as an exposure biomarker with a BEI® of 1.6 g/g of creatinine. Table 4 shows levels found in children in various studies outside the working and industrial environments in Latin-American countries and in areas with similar pollution problems. As pointed out by Bolden et al. [10], levels found are generally lower than those set forth in international reference values, like the US-EPA or ACGIH, for occupational exposure, but effects on health or on the measured biomarker are reported.

**Table 4:** VOC exposure and health effects in children.

VOC studied	Place	Biomarker	Age	Health effects	Exposure concentrations	Reference

Benzene	Valencia, Spain, big city	Home outdoor and indoor levels	Infants	No conclusive relationship with respiratory illness was found	Outdoor levels: 0.5–3.61 µg/m^3^, i/o ratio: 0.3–26.08 µg/m^3^ (highest in winter)	[72]
BTEX, and other 37 VOC	Genoa, Italy, with petrochemical industry away from residential zone	Home outdoor levels	6–10 years old	BTEX did not differ between zones. Higher risk of respiratory syndromes and absenteeism in children living in the industrial zone	114.4 µg/m^3^ of total VOC in the industrial area vs. 80.7 µg/m^3^ in the residential area. Respiratory effects are attributed to o-xylene	[44]
Benzene, toluene, ethylbenzene, xylenes and formaldehyde	Viseu, Portugal, nonindustrial city	Forced expiratory volume (FEV), forced vital capacity (FVC). VOC levels indoors and outdoors	7.3 ± 1.1 years old	Toluene levels, ethylbenzene and benzene exposure increases were associated with the need for medical intervention related with wheezing	0.5–39.2 µg/m^3^ benzene levels in a year; 3.3–108.2 µg/m^3^ toluene levels; 0.6–60.6 µg/m^3^ ethylbenzene levels; 2.1–185.6 µg/m^3^ xylenes levels in a year	[40]
Benzene, toluene, xylenes, NO_2_	Guamaré, Rio Grande do Norte, Brazil. City with petrochemical industry	Air quality monitoring station and respiratory symptoms in children	0–14 years old	VOC were under acceptable levels. Significant associations were found between wheezing and the homes closer to highest VOC levels in air	Benzene levels 32.4 µg/m^3^; Toluene levels 18.8 µg/m^3^; Xylenes levels 18.1 µg/m^3^	[43]
BTEX and other VOC, alkanes and cycloalkanes	La Plata, petrochemical zone compared to heavy traffic and nonpolluted areas	Forced expiratory volume (FEV), forced vital capacity (FVC). VOC levels outdoors	6–12 years old	Children in the petrochemical area showed significantly more respiratory problems during the winter season, including asthma	Benzene levels 19.3 µg/m^3^; Toluene levels 19.1 µg/m^3^; Xylenes levels 9.6 µg/m^3^; Total VOC levels 102.1 µg/m^3^	[42]
Benzene, toluene	Guangzhou, China, industrial city	8-OHdG, *t,t*-muconic acid (*t,t*-MA), dihydroxybenzene, s-phenylmercapturic acid (S-PMA), s-benzylmercapturic	3–6 years old	Co-exposure to benzene and toluene, plus HAP, induces additional DNA damage	Levels of t,t-MA: 2.6 to 381.16 µg/g creatinine; sum of all toluene and benzene metabolites: 25.09 to 1175.15 µg/g creatinine	[51]
Benzene	Kinshasa, Democratic Republic of Congo. Urban area	Phenylmercapturic acid (S-PMA) in urine	1–5 and 6–14 years	S-PMA levels are 10X higher than those reported for Canadian kids. A level of 7.0 µg/L in urine is proposed as a reference value	S-PMA level of 7 µg/L urine was found as a geometric mean in children aged 6 to 14 years old	[35]
Benzene, toluene	Coatzacoalcos, Veracruz, México. Petrochemical and oil refinery city	*t,t*-muconic acid *(t,t-MA)* and hipuric acid (HA) in urine. Comet assay and hematologic parameters	6–12 years old	Toluene and benzene metabolites did not exceed occupational levels. t,t-MA inversely correlated with hematocrit and hemoglobin values, as well as with red cell count	44–5521 µg/g creatinine of t,t-MA; 0.03–2.12 g/g creatinine of HA	[50]
Benzene	Two urban-industrial areas in Buenos Aires, Argentina	*t,t*-muconic acid and benzene in air	7–11 years old		t,t-MA 48.6 to 1367.8 µg/g creatinine, and benzene levels of 0.04 to 0.49 mg/m^3^	[49]
Benzene, toluene	San Luis Potosí, México. Urban area	*t,t*-muconic acid and hipuric acid in urine	9.5 ± 4.0 (6–13.5) years old		*t,t*-MA levels of 35 to 850 µg/g creatinine; HA levels of 0.1 to 1.75 g/g creatinine. Under reference values for both exposures	[53]
VOC	San Luis Potosí, México. Indigenous communities close to polluted rivers in a rural neglected area	*t,t*-muconic acid and 1-hydroxy-pyrene in urine	Children aged 7.5 ± 1.5 years old		t,t, MA: 38.4–4334 µg/g creatinine; 1-OHP: 0.02–94.1 µmol/mol creatinine;	[52]
VOC of industrial and mobile sources	Atoyac River in Central México	Forty-two sampling stations in six zones along the river	All the population living in a strip of two kilometers measured from the banks of the river	They conclude that the loss of ecological equilibrium in the basin represents an immediate and future health risk for inhabitants on the banks	Concentrations were not disclosed; however, the river does not sustain macroscopic life	[73]
BTEX	Global-42 studies from around the world and several settings: industrial, urban, rural	Air levels, indoor, outdoor, personal and urinary metabolites	Prenatal to senescence	Developmental, immune function, reproductive, respiratory, hematological, cardiovascular disease	BTEX levels under reference values established by EPA	[10]

As a reference, ACGIH has recommended the following BEI for BTEX in occupational environment: benzene, S-PMA – 25 µg/g creatinine, t,t-MA – 500 µg/g creatinine; toluene, hippuric acid – 1.6 g/g creatinine, o-cresol – 0.3 mg/g creatinine.

Tuakuila et al. [35] used S-phenylmercapturic acid (S-PMA) to measure benzene exposure. This metabolite was proposed by the ACGIH in its 2015 publication as the best benzene exposure biomarker [36], which had rendered previous levels measured by t,t-MA questionable. However, Fang et al. [37] demonstrated that there is a good correlation between the two metabolites in experimental animals and in children exposed to benzene in an industrial area in Korea.

### BTEX and their relationship to diseases in children

Due to the interaction of metabolites resulting from biotransformation of compounds with macromolecules, one can assume that systems will be affected in different ways, contributing to the development of a diversity of diseases, of which a vast collection of research has been carefully reviewed by Bolden et al. [10] They reached the important conclusion that even though environmental levels of these compounds are below reference levels, they cause various diseases because of the known characteristics of each BTEX component. BTEX are distributed to the central nervous system, the bone marrow and highly vascular tissues rich in lipids and are biotransformed both in the respiratory system and in the liver [38,39,40,41], where biotransformation can generate different endpoints of toxicity at the cellular level (Figure 3).

Studies on environmental exposure largely found a relationship with diseases such as asthma and respiratory complications (Table 4) [40,42,43,44]. Studied populations are located close to a source of BTEX, including industrial complexes, petrochemical facilities or refineries or large cities.

A study was conducted on children in elementary schools, one near a refinery and the other in an area far from the petrochemical complex. A relationship was observed between respiratory syndromes and resulting absenteeism and BTEX levels, and a significant difference was found in p-, m-xylenes concentration, which was greater in the area close to the refinery and correlated with sore throat, cough and cold in the children within the community [44].

Another study estimated concentrations of VOCs, including benzene, toluene and ethyl-benzene, with daily activity patterns in a sample of school age children in a nonindustrial area. This study was correlated with measurements of the forced expiratory volume (FEV) and the forced vital capacity (FVC), with the use of spirometric tests to identify respiratory capacity problems, and it found that the increase in total exposure was significantly associated with a decrease in FEV. Consequently, they suggest that this type of exposure may be related to the occurrence of asthma [40].

Lopes de Moraes et al. [43] found an association between wheezing and the levels of VOC at home; whereas, Wichman et al. [42] found more respiratory problems during the winter season. Similar results were seen in elderly people when looking for associations between the loss of pulmonary function measured by FEV and FVC tests and BTEX exposure measured through BTEX metabolites in urine, and the results were correlated with oxidative stress biomarkers (MDA, TBA, 8-OHdG), demonstrating that exposure to toluene and xylene has harmful effects on pulmonary function and that oxidative stress could be involved in the pathogenesis of this population [39].

This result matches the result seen in a model of young rats that were simultaneously administered a dose of toluene, chloroform and methylene chloride for three days, which produced an increase in lipid peroxidation measured by TBARS, a loss in antioxidant capacity determined by SOD, glutathione peroxidase and glutathione reductase diminished activities, as well as an increase in CYP2E1 hepatic activity. Individual administration of each compound did not induce a significant response in the animals [45].

**Figure 4:**
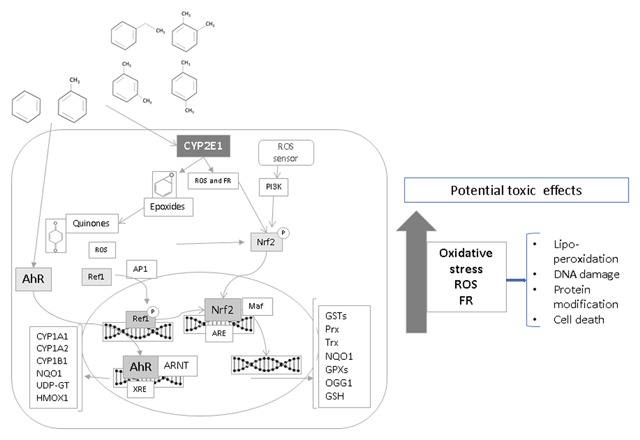
Oxidative stress, gene regulation and cytotoxic effects due to exposure to BTEX. ROS-reactive oxygen species; FR-free radicals. Prx – peroxiredoxin; Trx – thioredoxin; GPXs – glutathione peroxidase.

In this model, the liver and bone marrow were affected. All this is consistent with the reconstruction of events from different studies shown in Figure 4, which represents BTEX-induced effects of metabolism and antioxidant response, as well as possible cell-wide toxic effects. In their review of studies conducted on children and teenagers and at birth, Bolden et al. [10] presented the following as the main targets affected by these mechanisms: the respiratory system, the immune system, the reticuloendothelial tissue, the skin and the developing embryo.

## Discussion

While a large part of BTEX emissions into the atmosphere originate from biogenic processes, a considerable amount comes from anthropogenic sources that could be regulated more efficiently. In the European Union and the United States, VOC emissions have been reduced by regulating industrial processes, improving vehicle engines and reducing gasoline, solvents, paints and other sources. Nevertheless, reference concentrations (RfCs) developed by the EPA (Table 3) far exceed BTEX concentrations determined in all research presented in this review. RfCs are due to critical adverse effects caused by chronic exposure to pollutants. The last update of BTEX compounds was in 2012, and some references provided by the IRIS are before 1996.

This review has found many publications that associate concentrations below those used as reference for toluene, ethylbenzene and xylenes with illnesses classified as critical by the EPA-IRIS, including processes such as the increase in oxidative stress [39] that are precursor mechanisms of chronic diseases, such as cancer, and illnesses related to chronic inflammation, such as bronchitis [46], asthma [47] and atopy [48].

However, only six publications [35,49,50,51,52,53] quantified BTEX metabolites in children’s urine samples, and each one quantified different metabolites. Among them, Pelallo et al. [50] reported the highest levels of child exposure to benzene and toluene in three communities surrounded by petrochemical facilities in a tropical coastal region. Olmos et al. [49] reported high levels of exposure to benzene in the industrial area of a large capital city like Buenos Aires. The same results recur in similar studies on children living in polluted industrial areas in Korea [37] and Mexico. Children living close to industrial settings show higher levels of metabolites of benzene, which has been the most studied VOC as it is carcinogenic. Furthermore, Flores et al. [52] found a high level of exposure to benzene in children of a remote rural community crossed by a river polluted with industrial waste in a continental tropical zone. That the highest levels of benzene exposure were found in tropical areas could be due to the high temperatures, which allow the widest distribution of contaminants into the air.

It should be added that other carcinogenic VOCs were detected in MX: 1,3-butadiene and formaldehyde reach personal exposure levels that raise concerns about the health of exposed people [24].

Although the EPA-IRIS are collecting updated information for mixed and individual pollutants, countries that follow the guidelines thereof—criteria developed more than two decades ago—are still in use. This situation cannot be allowed to continue given the amount of information about BTEX biotransformation. It is now known that as a result of BTEX exposure, the body may form a variety of metabolites, such as epoxides and benzoquinones, that may harm macromolecules, which trigger the production of oxidative stress molecules and may become toxic because they irrupt into different routes of cell signaling, like cell differentiation, growth and death, and even mutate, all of which may result in the development of cancer cells.

In this regard, there is mounting evidence of the relationship between exposure to environmental pollution and acute myeloid leukemia in children, mainly from benzene exposure [54,55]. It has been shown that exposure to this compound induces a series of responses related to the cancer process [56], which may be strengthened by simultaneous exposure to other VOCs, as suggested by Belmont et al. [45], who showed a synergistic effect on oxidative stress induction from exposure to toluene, chloroform and dichloromethane. Cancer is just one of several effects that VOCs may have on health: it has been proven that they also affect smell perception, which seriously affects well-being, and that they produce behavioral changes by affecting the central nervous system [2].

Oxidative stress produced from exposure to BTEX may be one of the main mechanisms causing diseases like the loss of pulmonary function [41], skin problems [46,57] and immunological system disorders [58]. This occurs through the methylation of key genes, like CYP2E1 [59], or the activation of signaling routes that foster inflammation, like PI3K and SAPK, and genetic regulation given by Nrf2 and AhR transcription factors, which foster the transcription of genes involved in the response to xenobiotics and oxidative stress itself, including GST, NQO1, CYP2 A1, CYP2E1, HMOX1, HSP70. This could mean that people with prolonged exposure to low levels of BTEX will show chronic inflammation [60], primarily in the respiratory system, which is the main route of entry into the body.

Chronic exposure may affect other sensitive body tissues, like the bone marrow, as evidenced in the IARC monograph 109 [61], where carcinogenicity by outdoor air pollution was declared. This causes leukemia and central nervous system cancer in children exposed to benzene via automobile exhaust smoke or as a result of living in petrochemical areas or because their parents were exposed to gasoline or PM_2.5_. Mother exposure during pregnancy has escaped epidemiological studies establishing a relationship with childhood leukemia, and it would be advisable to consider this aspect, because it has been determined that childhood leukemia appearing in the early years of life may have originated during embryo development [62].

Problems with the study of BTEX lie in the fact that they are not the only pollutants in the atmosphere; thus, it is complex to attribute the effects found in an epidemiological study entirely to them. Exposure to industrial pollution, for example, usually includes exposure to metals as well.

Nevertheless, studies presented in this review show the global reach of VOC pollution, and it is hard for an individual not to be exposed to these compounds, even during embryo development. This type of exposure could also affect the fetal endocrine system, as suggested by Bolden et al. [10], thus altering not only the developing individual, but also the individual’s potential descendants. Therefore, the present environmental pollution situation already becomes a three-generation issue.

In a recent report by the Lancet Commission on Pollution and Health [63], deaths resulting from modern environmental pollution (due to toxics in the air and the occupational environment, as well as soil contamination) were estimated between five and six million worldwide in 2015. A significant aspect is that diseases caused by pollution are of a chronic-degenerative type, not communicable, in 71% of the cases. Major diseases found in children include diabetes, asthma, loss of cognitive function, attention deficit disorder or hyperactivity and autism.

Both the type of pollution and its sources vary as a country develops and industrializes, involving both a health and social cost that is reflected in economic losses of millions of dollars, equal to between 1.7% and 7% of a country’s annual healthcare spending, being higher in low and middle-income countries. It was calculated that welfare losses due to pollution amount to 4.6 trillion US dollars per year. These data emphasize the importance of implementing adequate and modern methods to register chronic diseases, particularly those occurring in polluted sites, to find the relationship between pollution and several illnesses that suddenly become frequent.

Such is the case of atopies – which have been highly recurrent in the last few years, especially in populations living in large cities or close to industrial complexes – and endocrine disruptive effects that alter the action of hormones. These findings are tools to demand better environmental policy regulations in several countries, both in the outdoor as well as the occupational environment, because neglecting the environment in the so-called emerging economies runs parallel to lax or completely absent hygiene measures at workplaces where air pollution could be reaching even higher levels than those reported here.

Under this regime, the predicted epidemic of cancer in developing countries is sadly turning into reality [64], particularly childhood leukemia, showing an increase of 21% in México from 2000 to 2004 compared to the levels from 1980 to 1984. México had the highest rate among Latin-American countries, with 5.76 per 100,000 deaths caused by leukemia among children aged 0 to 14 years [65].

## Final remarks

One action critical to keeping emissions into the environment below limits set by organizations like the EPA or ACGIH is implementing permanent environmental monitoring programs, much like in large cities, in populations near highly polluted sites: dumpsites, polluted rivers, industrial and petrochemical areas. A record should be made of every case of associated diseases like those described herein to implement effective health monitoring, which will allow for caring for and supporting these populations and their demands for fulfillment of environmental laws and human rights, because they have the right to breathe clean air, drink clean water and live in a healthy environment. These registers would also contribute to a better understanding of pollution health effects.

Developing countries would greatly benefit from the support of the World Health Organization looking at the domestic microenvironment and issuing recommendations related to environmental health. By resolving the microenvironment, the global environment will certainly improve, including global climate change, which is currently the focus of attention for developing countries. Ultimately, they could leave behind the environmental problems posed by proliferating industrial sites and the health and social issues they inevitably carry.

The experience of developed countries should be shared with developing countries so they can avoid the catastrophic consequences of industrialization and urbanization taking place supposedly to benefit their economies. As established by the Lancet Commission, “The claim that pollution control stifles economic growth and that poor countries must pass through a phase of pollution and disease on the road to prosperity has repeatedly been proven to be untrue” [63].
